# Comparing postdeposition reactions of electrons and radicals with Pt nanostructures created by focused electron beam induced deposition

**DOI:** 10.3762/bjnano.8.240

**Published:** 2017-11-15

**Authors:** Julie A Spencer, Michael Barclay, Miranda J Gallagher, Robert Winkler, Ilyas Unlu, Yung-Chien Wu, Harald Plank, Lisa McElwee-White, D Howard Fairbrother

**Affiliations:** 1 Department of Chemistry, Johns Hopkins University, Baltimore, MD, 21218, USA; 2 Graz Centre for Electron Microscopy, Steyrergasse 17, 8010 Graz, Austria; 3 Department of Chemistry, University of Florida, Gainesville, FL, 32611-7200, USA,; 4 Institute of Electron Microscopy and Nanoanalysis, Graz University of Technology, Steyrergasse 17, 8010 Graz, Austria

**Keywords:** atomic hydrogen, atomic oxygen, electron beam processing, focused electron beam induced deposition (FEBID), purification

## Abstract

The ability of electrons and atomic hydrogen (AH) to remove residual chlorine from PtCl_2_ deposits created from *cis*-Pt(CO)_2_Cl_2_ by focused electron beam induced deposition (FEBID) is evaluated. Auger electron spectroscopy (AES) and energy-dispersive X-ray spectroscopy (EDS) measurements as well as thermodynamics calculations support the idea that electrons can remove chlorine from PtCl_2_ structures via an electron-stimulated desorption (ESD) process. It was found that the effectiveness of electrons to purify deposits greater than a few nanometers in height is compromised by the limited escape depth of the chloride ions generated in the purification step. In contrast, chlorine atoms can be efficiently and completely removed from PtCl_2_ deposits using AH, regardless of the thickness of the deposit. Although AH was found to be extremely effective at chemically purifying PtCl_2_ deposits, its viability as a FEBID purification strategy is compromised by the mobility of transient Pt–H species formed during the purification process. Scanning electron microscopy data show that this results in the formation of porous structures and can even cause the deposit to lose structural integrity. However, this phenomenon suggests that the use of AH may be a useful strategy to create high surface area Pt catalysts and may reverse the effects of sintering. In marked contrast to the effect observed with AH, densification of the structure was observed during the postdeposition purification of PtC*_x_* deposits created from MeCpPtMe_3_ using atomic oxygen (AO), although the limited penetration depth of AO restricts its effectiveness as a purification strategy to relatively small nanostructures.

## Introduction

Focused electron beam induced deposition (FEBID) has demonstrated great potential in the field of nanostructure fabrication [1–4]. In FEBID, a volatile organometallic precursor is introduced into a vacuum chamber (typically a modified scanning electron microscope (SEM)) and irradiated by a focused electron beam [2–3]. The precursor decomposes under electron beam irradiation, with non-volatile product species being incorporated into the growing deposit. The size and shape of the nanostructure are controlled by manipulation of the electron beam (focusing ability and patterning capabilities), which allows an almost unlimited array of three-dimensional nanostructures to be fabricated.

Despite the significant advantages of a process that can direct write nanostructures without the need for resists or masks [2–3], FEBID has several challenges preventing its wider implementation as a robust method for nanofabrication. One of the biggest issues is deposit purity [5]. FEBID deposits often contain <30% metal content [2,5], and as a result, methods for the purification of FEBID structures have recently been explored [5–13]. However, some purification approaches that remove chemical impurities and decrease metal content negatively impact the shape integrity of the deposits by producing voids, cracks or other unwanted side effects [5,9–10,14–15]. An ideal purification strategy in FEBID is one that removes all of the organic impurities to leave behind a compact, high-fidelity metal nanostructure, whose shape is unchanged as compared to the as-deposited structure. Consequently, approaches which have been attracting increased interest are the so-called “low temperature” purification strategies, where carbon is removed at temperatures low enough to avoid changing the structure and morphology of the deposit.

In FEBID purification studies, the removal of carbon is often emphasized due to its prevalence as an impurity in the deposited nanostructures. One of the most widely studied non-thermal purification strategies is electron beam induced purification, typically performed in the presence of a gas-phase species, usually either oxygen or water. In these techniques, the electron beam dissociates gas phase reactants to yield reactive oxygen species, which then convert deposited carbon into volatile compounds such as CO and CO_2_ [16–19]. Villamor et al*.* [20] observed that either by post deposition electron beam processing in the presence of O_2_ gas, or by using both precursor and O_2_ gas simultaneously during deposition, nearly 100 atom % Pt deposits could be generated from FEBID nanostructures created from the commonly used precursor MeCpPtMe_3_. Simultaneous deposition and etching produced void-free structures with resistivity only six times greater than pure Pt metal. In a study by Lewis et al. [21], FEBID nanostructures created from MeCpPtMe_3_ were subsequently purified by electron irradiation in the presence of oxygen and examined by cross-sectional SEM, which revealed that purification occurred in a top-down fashion. Mansilla et al. [22] developed a novel concentric nozzle for FEBID that allows for in situ O_2_ purification during deposition. This approach was used to purify deposits created from Me_2_Au(acac) and resulted in an order of magnitude decrease in the C/Au ratio and orders of magnitude improvement in resistivity. Mehendale et al. [14] also observed that high purity Au nanostructures (C/Au < 0.2, compared with C/Au = 0.06 for pure bulk Au) could be generated using electron beam postprocessing in the presence of O_2_ with minimal shape distortion.

Carbon atoms can also be removed by electron beam purification using H_2_O. Geier et al. [13] demonstrated that for FEBID structures created from MeCpPtMe_3_, postdeposition electron beam irradiation in the presence of a local water pressure of 10 Pa results in a highly efficient electron-limited etching regime. This process enabled purification rates of better than 5 min nA^−1^μm^−2^. The results were consistent with extremely fast inward diffusion of the water molecules through the carbon matrix, after which the incorporated water was dissociated by electron irradiation to produce reactive oxygen species. Cross-sectional TEM data revealed that purification does not occur in a top-down manner, but is rather controlled by the penetration depth of the incident electron beam. At a beam energy of 5 keV, complete carbon removal could be obtained up to an initial thickness of 150 nm. In addition to purification, the purified deposit was compacted to form a high-fidelity, pore-free array of Pt atoms in which the original shape of the deposit was retained with little morphological change. Shawrav et al. [23] demonstrated the effectiveness of water in the purification of Au nanostructures. The single-step fabrication of highly pure Au nanostructures (≈91 atom % Au) from Me_2_Au(tfac) with co-deposition of water vapor resulted in Au FEBID nanostructures with the highest conductivity achieved to date (resistivity of 8.8 μΩ cm, compared with 2.2 μΩ cm for pure Au [12]).

Another recent purification method is laser-assisted electron beam induced deposition (LAEBID) [24], where purification is ascribed at least in part to a laser-induced oxidation process. In this technique, the reactive oxygen species are produced from gas phase reactants, such as oxygen, that are deliberately introduced. Sequential cycles of electron-induced deposition are followed by laser-induced, spatially localized annealing (producing a temperature increase on the order of 300–400 K). Using this approach, Stanford et al. [24] reported that LAEBID augmented by reactive gases (O_2_) decreased the C content by 75% in nanostructures created from MeCpPtMe_3_. In related work, Lewis et al. [25] found that purification by LAEBID resulted not only in higher platinum content but also in an improved platinum coalescence and a transition from amorphous to graphitic carbon. The net effect of these chemical and structural transformations was a 100-fold improvement in nanowire resistivity, while maintaining a high degree of nanostructure resolution.

Low-temperature purification can also be achieved by reactive species generated by an independent source. Botman et al. [26] treated FEBID deposits created from MeCpPtMe_3_ with atomic hydrogen (AH), which resulted in a decrease in carbon content (from 81 to 65 atom %) with no deposit damage or void formation. In a Cu example, Miyazoe et al. [27] investigated H_2_–Ar microplasma effects on FEBID deposits created from Cu(hfac)_2_. Postgrowth purification resulted in an increase in Cu content from ≈12% to 27%, coupled with a volume decrease and an increase in surface roughness. Wnuk et al. [28] subjected deposits created from Me_2_Au(acac) to AH and/or atomic oxygen (AO). AH removed all of the O atoms and the majority of C atoms from the deposit while AO removed all of the C atoms far more efficiently than AH, but with some accompanying Au oxidation. However, exposure to a sequence of AO followed by AH resulted in purely metallic Au, with AFM studies showing evidence that purification was accompanied by a decrease in deposit size. In the present study, the effect of AO on deposits created from MeCpPtMe_3_ has been investigated and compared to previous results on postdeposition electron-induced purification methods using O_2_ and H_2_O [13,17].

The majority of the work described in this study investigates potential strategies for purifying platinum-based FEBID deposits created from *cis*-Pt(CO)_2_Cl_2_ by treatment with electrons and AH, strategies which were deemed capable of removing chlorine from these deposits. We have previously shown that *cis*-Pt(CO)_2_Cl_2_ affords deposits that contain only Pt and Cl atoms [29], which hereafter will be referred to as PtCl_2_ deposits. In contrast to the extensive work on removing carbon from deposits, there is a general lack of information on postdeposition purification strategies capable of removing halogen atoms, despite the presence of halide ligands in many organometallic precursors. Compared to carbon, contaminant halogen atoms in FEBID structures present a different challenge when it comes to purification strategies as they cannot be removed by reactions with reactive oxygen species (ROS) to create volatile species such as the CO and CO_2_ compounds formed from carbon impurities. It should be noted that the use of halogen-containing precursors must always be viewed with caution [2,30] due to the potential of gas phase halogen-containing species to etch substrates or equipment.

The use of electrons was motivated in part by previous ultrahigh vacuum (UHV) surface science studies which showed that for 1–2 monolayer (ML) thin films of organometallic precursors with halide ligands, the halogens can be removed [29,31]. The importance of halogen desorption initiated by electron irradiation in FEBID is also supported by the observation that FEBID nanostructures created from WF_6_, WCl_6_ and SiH_2_Cl_2_ contain (W/Si):halogen ratios far greater than those of the precursor molecules [32–33]. Indeed, in the case of SiH_2_Cl_2_, electron beam irradiation of an adlayer of SiH_2_Cl_2_ resulted in an exponential decay of the Cl signal, until it was indistinguishable from the background level signal [33]. In more recent work, with halogenated precursors, highly pure Au nanostructures (>95 atom %) have been created from both PF_3_AuCl [34] and AuCOCl [35] with no Cl present in the deposits, implying efficient electron-stimulated desorption/loss mechanisms during deposition. Similarly, van Dorp et al. [36] recently found that FEBID deposits produced from the Au(III) dimer (ClAuMe_2_)_2_ are almost completely free of Cl (2–6 atom %). Studies with AH were motivated by previous reports [37] which have shown that AH can react with adsorbed halogen atoms in an Eley–Rideal-type process [38–42] to directly remove halogen atoms from surfaces in the absence of thermal equilibration. It is also possible that the formation of AH contributes to the effectiveness of a postdeposition purification strategy where FEBID structures created from Co_2_(CO)_8_ were annealed to 300 °C and exposed to H_2_ and electron irradiation leading to the formation of compact, carbon- and oxygen-free 20 nm thick Co layers [43]. As a means of comparison, the effect of AH on other contaminant elements present in the platinum-containing precursors (MeCpPtMe_3_, Pt(hfac)_2_, and Pt(PF_3_)_4_) was also evaluated.

## Experimental

FEBID structures were fabricated using two different systems: (i) a PHI 610 scanning Auger microprobe system (Auger electron spectroscopy (AES)), where deposits were subsequently treated either in situ with electrons or ex situ using AH, and (ii) a FIB Nova 200 dual beam microscope, where deposits were exposed ex situ to AO.

### Deposition, characterization and treatment of FEBID structures using Auger electron spectroscopy

Details of the Auger electron spectroscopy (AES) chamber and its analytical capabilities can be found in previous publications [8,28,44]. The precursor *cis*-Pt(CO)_2_Cl_2_ was synthesized as previously reported [29]. The remaining Pt-containing compounds, trimethyl(methylcyclopentadienyl)platinum (MeCpPtMe_3_) (CAS 94442-22-5, Sigma-Aldrich), platinum hexafluoroacetylacetonate (Pt(hfac)_2_) (CAS 65353-51-7, Strem Chemicals, Inc.), and tetrakis(trifluorophosphine)platinum (Pt(PF_3_)_4_) (CAS 19259-53-4, Strem Chemicals, Inc.), were purchased and used as received.

The deposits were created by introducing each of the precursors into the vacuum chamber through a UHV-compatible leak valve which was attached to a directional doser. To maintain a sufficient vapor pressure of *cis*-Pt(CO)_2_Cl_2_ during deposition, this precursor was heated to ≈80 °C [29]. The other precursors (MeCpPtMe_3,_ Pt(hfac)_2_, and Pt(PF_3_)_4_) were sufficiently volatile for deposition to proceed without heating the precursors [44–46]. The electron beam of the PHI 610 scanning Auger microprobe system (LaB_6_ filament) was used in three ways: 1) as the electron source for deposition, 2) to characterize deposit elemental composition by AES, and 3) to conduct electron beam postprocessing. For each of these studies the electron beam characteristics were as follows: beam voltage of 3 kV, average target current 300 nA, beam shape ≈10 × 50 µm, where the latter is defined by the size of the deposits. Each of the deposits was created with a pressure of P*_cis_*_-Pt(CO)2Cl2_ ≈ 1.5 × 10^−8^ Torr for 23 h. Atomically smooth, Ru-capped, Si/Mo multilayer mirror substrates [47] were used for most depositions, although highly ordered pyrolytical graphite (HOPG) and SiO_2_ substrates were used for a few depositions. The Ru-capped, Si/Mo multilayer mirror substrate was preferred due to the smoothness and ease with which deposits could be identified and imaged by SEM. All of the effects of electrons and atomic hydrogen reported in this study were independent of the substrate on which the deposits were created.

Deposits generated in the Auger system were imaged and analyzed ex situ using a cold-cathode field-emission SEM (JEOL 6700F, LEI detector) with 1.0 nm resolution at 15 keV equipped with an energy-dispersive X-ray analyzer (EDS Genesis 4000 X-ray analysis system, detector resolution of 129 eV). Unless otherwise noted, SEM images were acquired with a beam energy of 10 keV. For a few experiments, it was necessary to deconvolute EDS spectral interferences (for Pt M and P K lines), which was done using a JEOL JXA-8600 superprobe SEM equipped with wavelength dispersive spectroscopy (WDS) capabilities. Previous data collected using the AES system has shown that deposits created from *cis*-Pt(CO)_2_Cl_2_ are composed exclusively of Pt and Cl [29]. Consequently, all EDS data are reported in terms of the Pt and Cl signals, ignoring small contributions from substrate peaks (e.g., Mo and Si).

### Generation of atomic hydrogen radicals (high pressure)

The majority of AH purification was conducted ex situ using a custom-built AH cleaning system located at the National Institute of Standards and Technology (NIST). This source passed H_2_ over a heated tungsten filament to produce a constant flux of AH radicals. The purification system at NIST permitted high-pressure H_2_ gas to be admitted (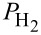 ≈ 1 Torr), resulting in a correspondingly large flux of AH radicals. During purification the sample surface was perpendicular to the AH source at a working distance of ≈3.8 cm.

### Generation of atomic hydrogen radicals (low pressure)

A much lower flux of AH radicals (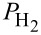 ≈ 5 × 10^−7^ Torr) was produced in situ in the Auger spectrometer with a thermal gas cracker (Oxford Applied Research) as described in previous publications [28,45,48]. During purification the sample surface was roughly perpendicular to the AH source at a working distance of approximately 5 cm. AH exposure is reported in terms of pressure and time as well as in units of langmuir (L), where 1 langmuir (L) = 10^−6^ Torr·s.

### Atomic force microscopy

Deposits created by FEBID from *cis*-Pt(CO)_2_Cl_2_ were imaged before and after AH cleaning by atomic force microscopy (AFM) in noncontact mode with a 75 ± 15 kHz HQ:NCS18 probe (Mikromasch USA, Watsonville, CA) on a PicoSPM SE AFM. Image processing of line-by-line leveling, surface roughness, profile extraction and 3D rendering was carried out with Pico Image Basic 5.0.2 software.

### Deposition, characterization and treatment of FEBID structures in the FIB Nova 200 system

In these experiments, FEBID was performed on a FIB Nova 200 dual beam microscope (FEI, The Netherlands) using a standard FEI gas injection system for delivering MeCpPtMe_3_. The precursor was heated to 45 °C for at least 2 h and the gas valve was opened at least 3 min prior to the deposition. Nine 5 × 5 µm^2^ Pt–C pads were deposited at a primary energy of 5 keV and a beam current of 1600 pA in a serpentine patterning sequence. A point pitch of 26 nm and a dwell time of 250 µs were used to ensure a flat-top deposit shape [49]. Nine different deposition heights, ranging from 14 to 73 nm were achieved by a variation of loops (1–9 loops), resulting in a variation of the total exposure times (TET) per pixel. The deposits were created on a 1 × 1 cm² silicon wafer (3 nm surface oxide) and spaced 5 µm apart from one another. The height and roughness characterization was done with via AFM (FastScan Bio, Bruker AXS, USA) in tapping mode and postprocessed with Gwyddion 2.44 software.

### Generation of atomic oxygen

FEBID structures generated in the Nova 200 were exposed to AO produced with the same thermal gas cracker (Oxford Applied Research) used in the low-pressure AH studies [28,45,48]. During purification the sample surface was roughly perpendicular to the source at a working distance of approximately 3 cm. The in situ AO purification was carried out at a pressure of *P*_O2_ ≈ 1 × 10^−6^ Torr over a period of several days.

## Results

### Postdeposition processing/purification of PtCl_2_ deposits

#### Electrons

**Figure 1:**
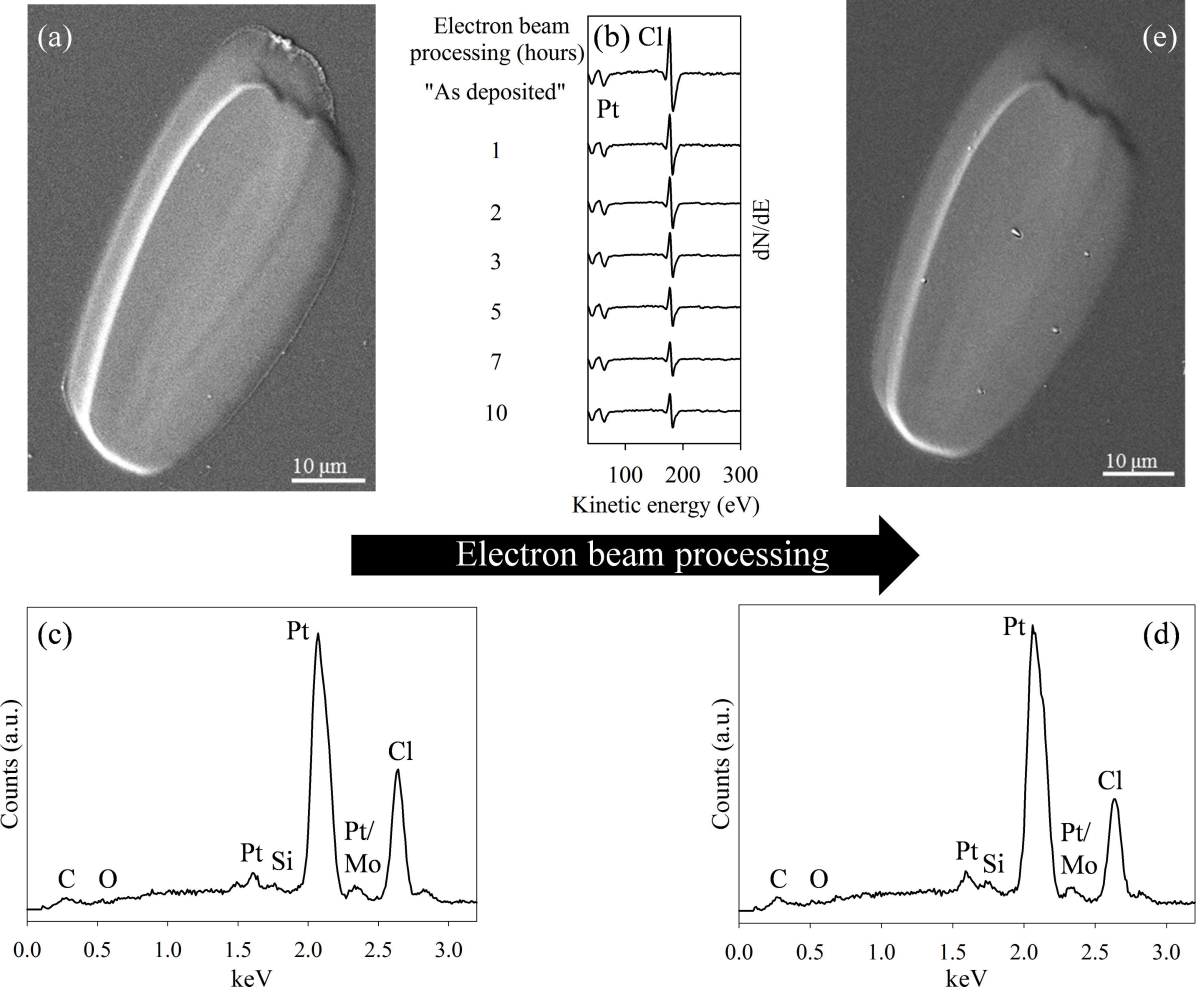
(a) Representative FEBID deposit created from *cis*-Pt(CO)_2_Cl_2_. Representative AES and EDS of “as-deposited” structures prior to any postdeposition electron beam processing are shown in (b) and (c); (b) also shows the evolution of the AES spectra as a function of electron exposure time. In (d) the EDS of a PtCl_2_ deposit after 20 h of postdeposition electron exposure is shown, as well as the corresponding SEM image in (e). The small C, O, Si and Mo peaks observed in EDS can be ascribed to the substrate (see Supporting Information File 1, Figure S2).

Figure 1a shows an SEM image of a PtCl_2_ deposit created from *cis*-Pt(CO)_2_Cl_2_ that contains only Pt and Cl as determined by analysis in the AES system and by EDS (Figure 1b,c). Based on the attenuation of the substrate peaks in EDS, and using an estimated penetration depth of ≈200 nm for the 10 keV electron beam together with the software package CASINO v2.48 [50], the PtCl_2_ deposits studied in this investigation are estimated to be at least 200 nm thick.

Figure 1b shows the effects of in situ electron beam processing of a PtCl_2_ deposit, as measured by AES. During the first 7 h of electron beam irradiation, the Cl atom % decreased significantly, leading to a concomitant increase in Pt atom % (from an initial value of 36% Pt to 56% Pt). However, for electron irradiation times in excess of 7 h, the Pt atom % remains relatively constant at ≈55–59% (see Supporting Information File 1, Figure S1a). In contrast, when a much thinner FEBID deposit (deposition time of 5.25 h compared with 22 h) was postprocessed by electron irradiation, the degree of purification was 87% Pt , as measured by AES (see Supporting Information File 1, Figure S1b). In contrast to the extremely surface-sensitive compositional changes observed in the AES (Figure 1b), EDS analysis (Figure 1d) shows that after 20 h of postdeposition electron beam processing in the Auger spectrometer, the PtCl_2_ deposits exhibited almost no change in the Pt atom %. Similarly, the Pt atom % determined by EDS remained relatively unchanged when a PtCl_2_ deposit was exposed to electron irradiation in the SEM for 2 h (data not shown). SEM images of electron beam irradiated PtCl_2_ deposits, such as the one shown in Figure 1e, appeared largely unchanged compared to the “as-deposited” structure. For EDS spectra, contributions from C, O, Si, and Mo are due to the substrate. Supporting Information File 1, Figure S2 provides a reference spectrum for the substrate as compared to the as-deposited PtCl_2_ deposit and a purified PtCl_2_ deposit.

#### Atomic hydrogen (AH)

**Figure 2:**
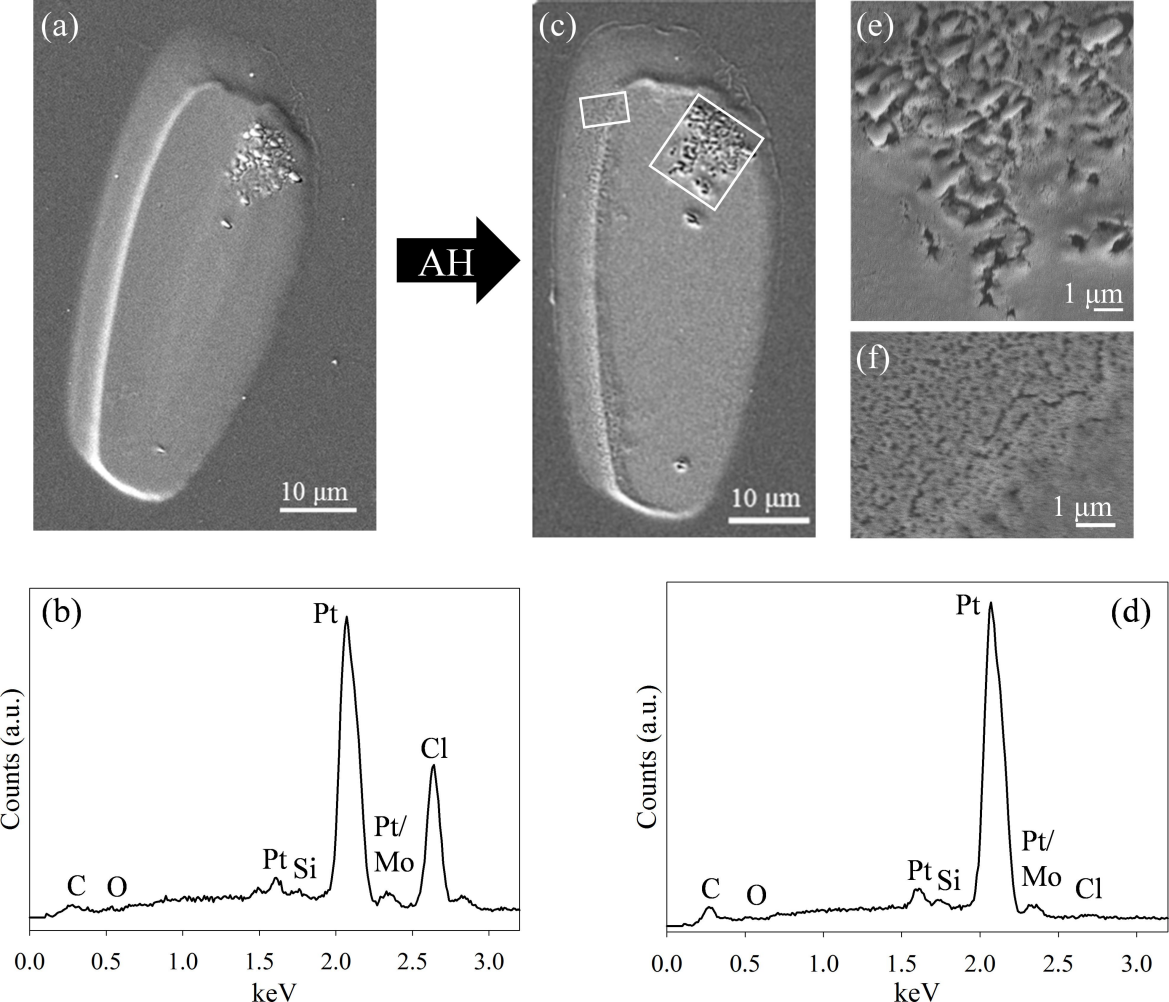
(a) SEM image of a deposit created from *cis*-Pt(CO)_2_Cl_2_, and (b) corresponding EDS spectrum. (c) SEM image and (d) EDS spectrum of the same deposit after exposure to 2 h of atomic hydrogen at 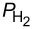 ≈ 1 Torr (7.2 × 10^9^ L). The white boxes in (c) denote locations where the magnified images shown in (e) and (f) were acquired.

**High-pressure AH source:** Figure 2 shows the effect of exposing a PtCl_2_ deposit to 2 h of AH at *P*_H2_ ≈ 1 Torr (7.2 × 10^9^ L). In the SEM images, the deposit appears to be unchanged in size and shape (compare Figure 2a and Figure 2c), although the pockmarked imperfections (predominant in the upper right corner) have become larger and there is some evidence of granularity, particularly at the edges (see Figure 2e,f). In terms of chemical transformation, a comparison of representative EDS spectra before and after exposure to AH (Figure 2b and Figure 2d) reveals that essentially all of the Cl has been removed and the deposit is now almost 100% Pt.

**Figure 3:**
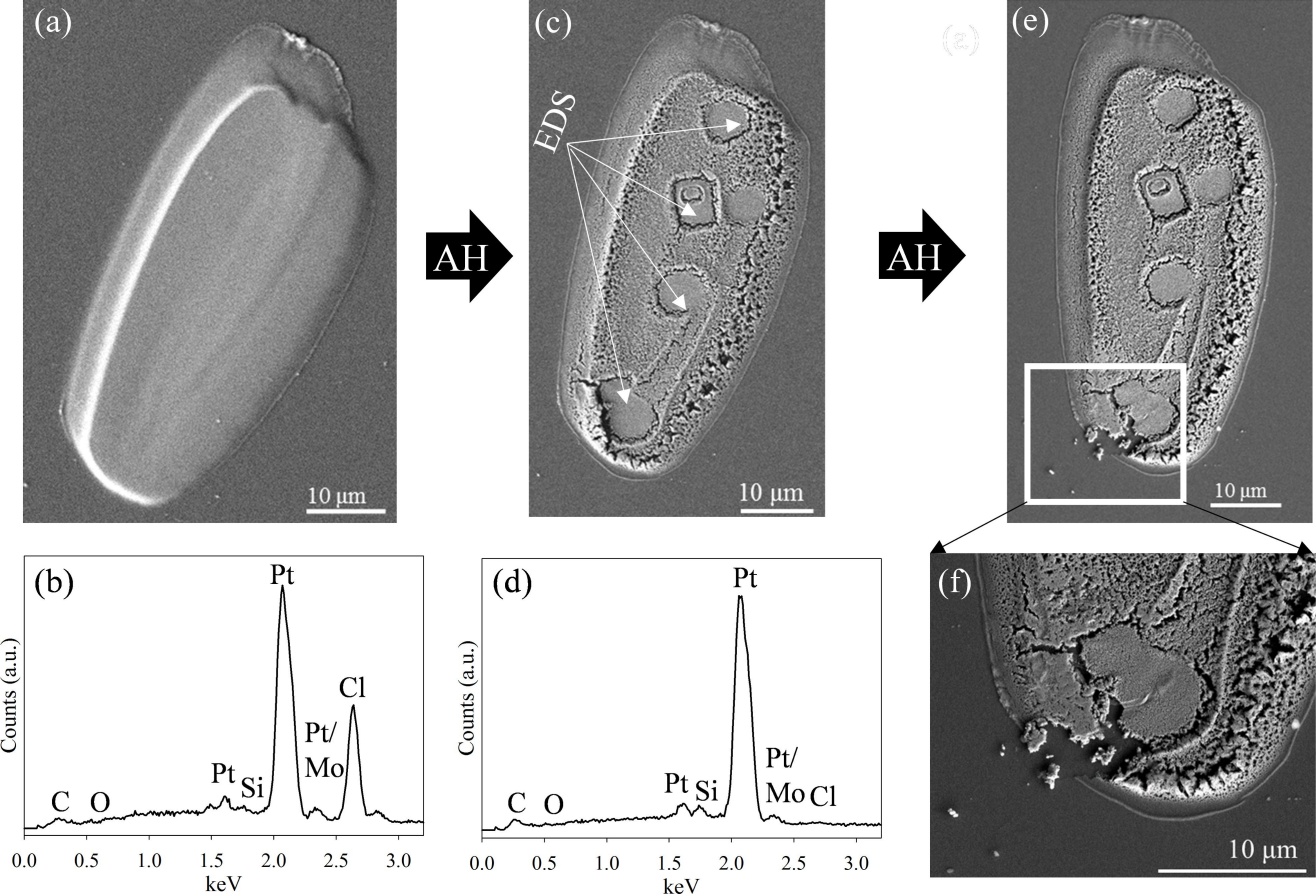
(a) PtCl_2_ deposit created from *cis*-Pt(CO)_2_Cl_2_; (b) corresponding EDS analysis. (c) SEM image of the deposit after 2 h of atomic hydrogen treatment at *P*_H2_ ≈ 1 Torr (7.2 × 10^9^ L), with arrows denoting areas where EDS analysis was initially performed. The EDS spectrum of the deposit after 2 h of AH exposure is shown in (d). The SEM image shown in (e) was taken after two additional hours of AH treatment (4 h total), with a magnified image of the lower left corner shown in (f).

Figure 3 shows the effect of exposing another PtCl_2_ deposit to 2 h of AH at *P*_H2_ ≈ 1 Torr (7.2 × 10^9^ L) under the same conditions. Consistent with the EDS data shown in Figure 2, all of the chlorine atoms have again been removed. However, although the shape of the deposit remains unchanged, the surface morphology has changed significantly, with a large amount of granularity and increased porosity, giving it a honeycombed appearance. Interestingly, in regions exposed to electron beam irradiation during EDS characterization of the initial deposit, little or no structural changes were observed (see also Supporting Information File 1, Figure S3). It should be noted that in this deposit, EDS analysis was acquired after approximately 5 min of electron exposure. In contrast, for other PtCl_2_ deposits reported in this study, EDS data was acquired after approximately 45 s of electron exposure. In these cases the initial location of the EDS analysis could not be discerned after AH exposure.

Figure 3e shows the structural evolution of the deposit shown in Figure 3c after two more hours of AH exposure (7.2 × 10^9^ L). Although most of the deposit is unchanged, the highlighted region (Figure 3f) shows that small (≤1 µm) Pt fragments have detached from the main structure in one area.

**Figure 4:**
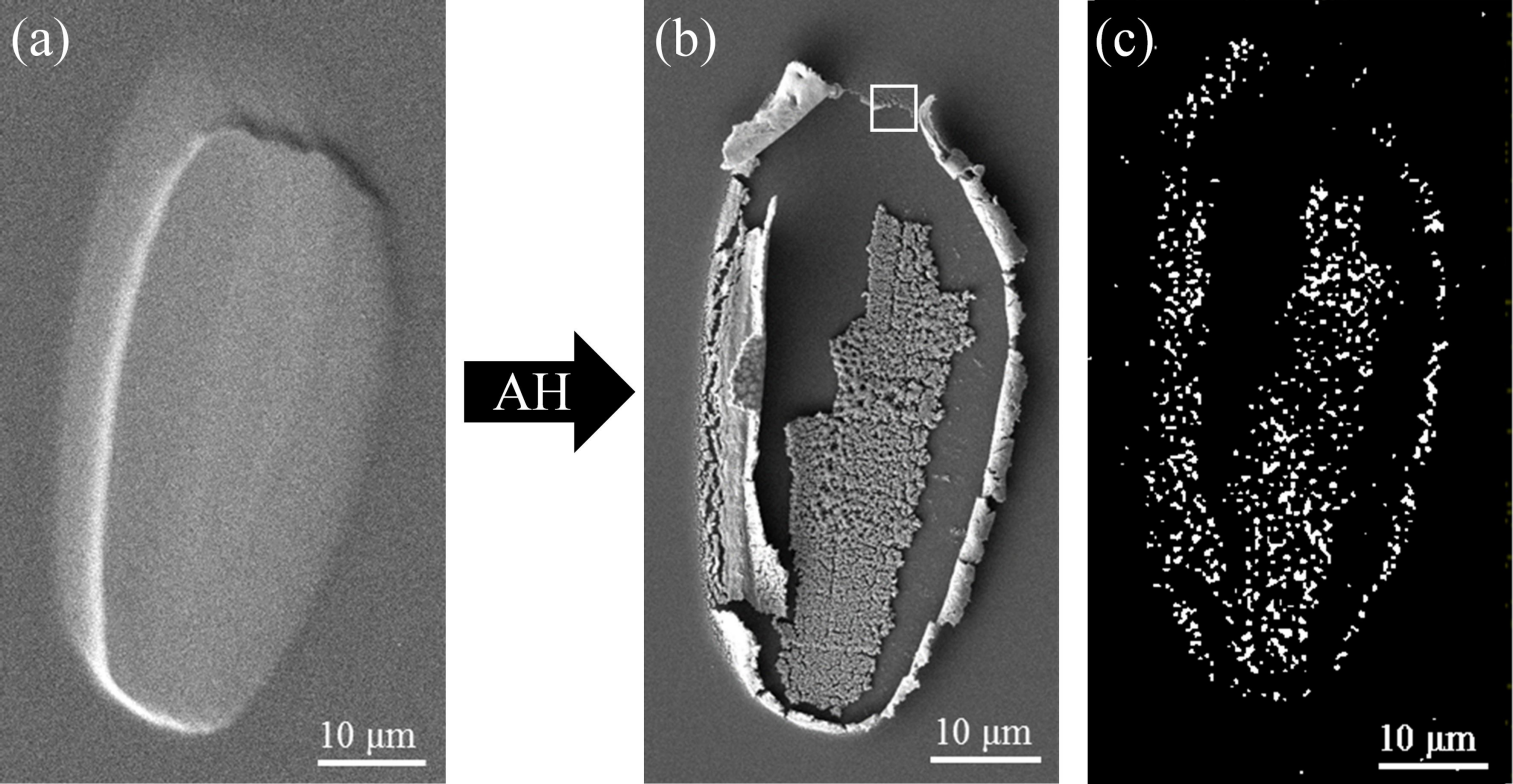
(a) SEM image of a PtCl_2_ deposit created from *cis*-Pt(CO)_2_Cl_2_, without any EDS analysis and prior to any atomic hydrogen treatment; (b) the deposit after two hours of atomic hydrogen treatment at *P*_H2_ ≈ 1 Torr (7.2 × 10^9^ L), with a corresponding EDS elemental map of Pt shown in (c). The highlighted area in (b) indicates the area of AFM analysis (see Supporting Information File 1, Figure S4).

Figure 4 shows the effect of two hours of high-pressure AH treatment (7.2 × 10^9^ L) on another PtCl_2_ deposit. In this case, exposure to AH not only removed all of the Cl atoms but also resulted in a complete and dramatic loss of structural integrity as shown in Figure 4b. The Pt EDS map (Figure 4c) reveals that the residual Pt atoms are present either as loosely packed structures in the center of the deposit or in the bands along the perimeter, with no Pt observed in between. The periphery of this deposit (see highlighted area in Figure 4b) was also analyzed by AFM (shown in Supporting Information File 1, Figure S4), with a line scan showing the atomically smooth nature of the Mo/Si substrate as well as the presence of an ≈100 nm high feature which can reasonably be assumed to be purified Pt.

**Figure 5:**
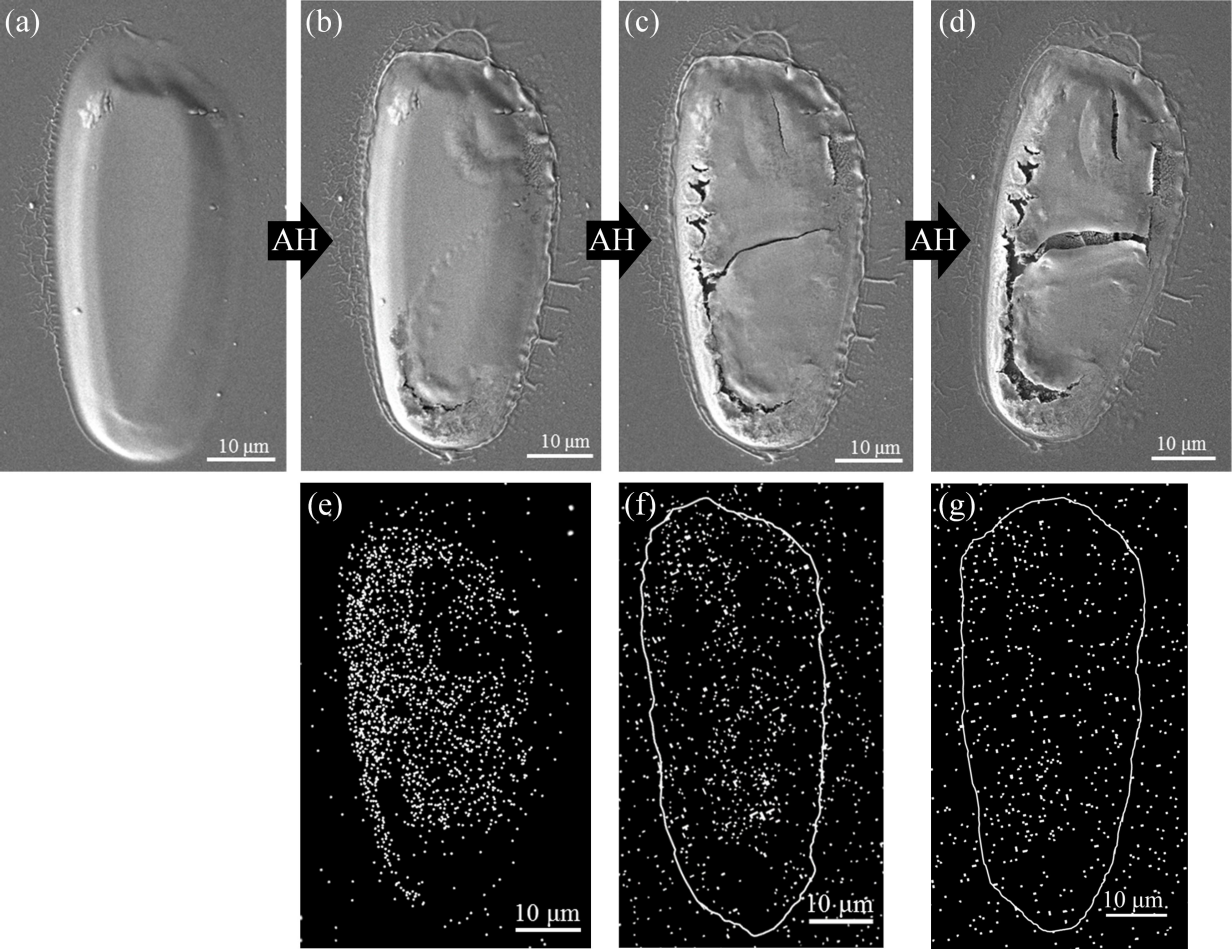
SEM images of a deposit created from *cis*-Pt(CO)_2_Cl_2_: (a) prior to any treatment, and after (b) 48, (c) 78 and (d) 198 min of AH exposure, corresponding to 2.9, 4.7 and 11.9 × 10^9^ L, respectively. The corresponding Cl EDS maps after (e) 48, (f) 78 and (g) 198 min of AH exposure are shown underneath. The white outlines drawn in (f) and (g) are a guide to the eye to show the location of the deposit.

Figure 5 shows the evolution of a PtCl_2_ deposit as it was exposed to increasing doses of AH from the high-pressure source. After this deposit was first exposed to the output of the atomic radical source without any hydrogen flowing, the chemical composition and structure of the deposit were unchanged, demonstrating that all of the effects shown in Figures 2–5 (and Supporting Information File 1, Figures 3 and 4) are caused by AH. This deposit was then subjected to successively larger AH exposures. At the end of each exposure, SEM images and EDS spectra were obtained. Once structural changes were macroscopically apparent, EDS mapping of the deposit was also performed. Figure 5a shows an SEM image of the structure as-deposited; after 48 min of AH exposure (2.9 × 10^9^ L) some cracks (lower left-hand section of the deposit) and undulations (upper right-hand region of the deposit) are observed in Figure 5b. The corresponding Cl EDS map (Figure 5e) reveals that these structural transformations correlate with regions of greatest Cl loss (<13 atom % Cl remained in lower left-hand region where the cracking was observed, while in the smoother regions >52 atom % Cl remained by EDS). When the AH exposure was increased to 78 min (4.7 × 10^9^ L) the structural transformations observed in Figure 5b propagate and become more apparent, continuing to track with regions of significant Cl removal, as shown in Figure 5c,f. Continued exposure of this deposit to AH resulted in further increases in the amount of “cracking” and changes in structural integrity. This was accompanied by a progressive loss of intensity in Cl K EDS maps (Figure 5f), although Pt M EDS maps remained largely unchanged (data not shown). After 198 min of AH exposure (11.9 × 10^9^ L), the majority of Cl had been removed and no further changes occurred (Figure 5d,g).

High-pressure AH purification was also evaluated using deposits from other Pt-containing precursors (MeCpPtMe_3_, Pt(hfac)_2_, and Pt(PF_3_)_4_) that do not contain chloride ions. In each case 2 or 2.5 h of AH exposure at *P*_H2_ ≈ 1 Torr (7.2 and 9.0 × 10^9^ L) was found to have little or no effect on deposit structure as determined by SEM (see Supporting Information File 1, Figures S5–S7) and the chemical composition as determined by EDS or WDS for Pt(PF_3_)_4_ (Table 1)).

**Table 1:** Purification data for other Pt-containing compounds, as-deposited and after atomic hydrogen treatment (2 h for MeCpPtMe_3_ and Pt(PF_3_)_4_, 2.5 h for Pt(hfac)_2_, 7.2 and 9.0 × 10^9^ L). The data were obtained from EDS measurements of MeCpPtMe_3_ and Pt(hfac)_2_ and from WDS measurements of Pt(PF_3_)_4_. Note that EDS contributions from the substrate have been ignored in Table 1 so that the data focuses only on changes to the deposit.

Precursor		

MeCpPtMe_3_	% Pt	% C

As-deposited	13.7	86.3
Post-AH treatment	13.3	86.7

Pt(hfac)_2_	% Pt	% C

As-deposited	27.2	72.7
Post-AH treatment	30.4	69.6

Pt(PF_3_)_4_	% Pt	% C

As-deposited	79.5	20.5
Post-AH treatment	79.3	20.7

**Figure 6:**
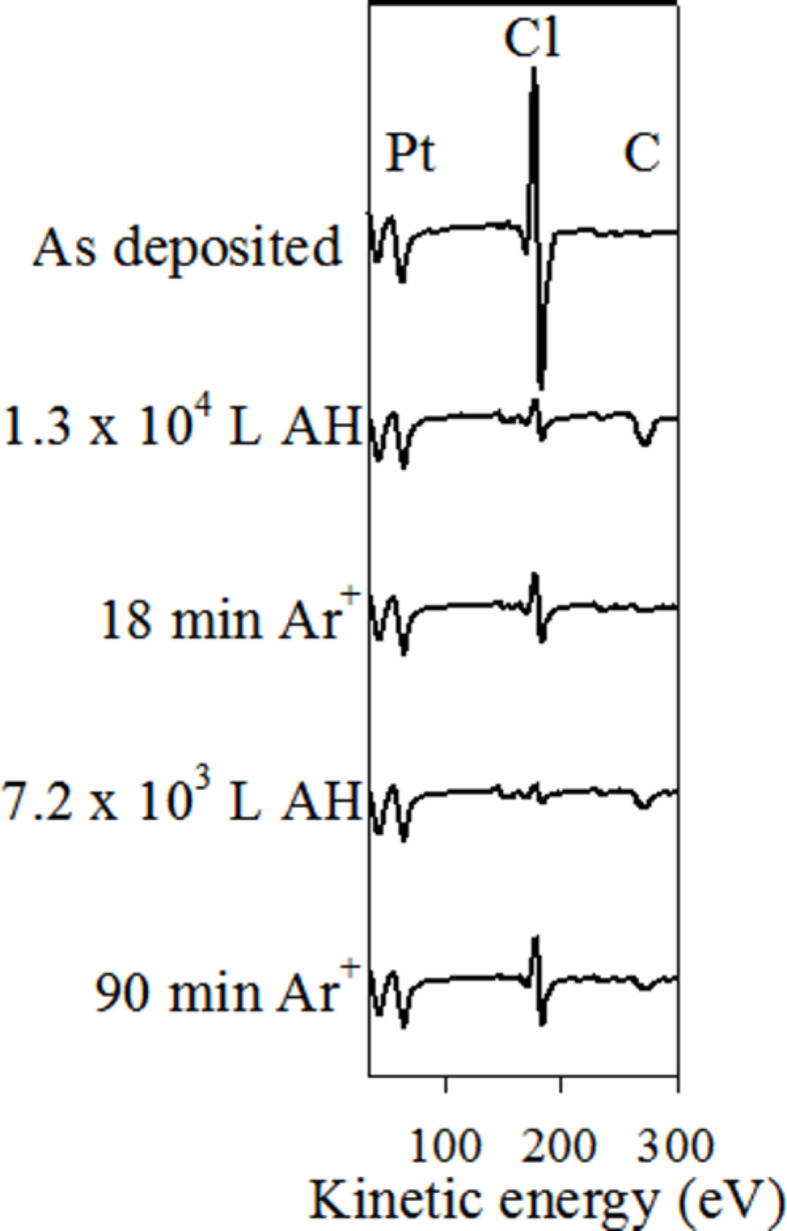
AES for a deposit created from *cis*-Pt(CO)_2_Cl_2_ (top spectrum) and then subjected to alternating treatments with atomic hydrogen (*P*_H2_ ≈ 5 × 10^−7^ Torr), and argon ion sputtering (*P*_Ar_ ≈ 5 × 10^−8^ Torr, 2 keV Ar+ ions).

**Low-pressure AH source:** Figure 6 displays the effect of AH generated by a low-pressure thermal gas cracker (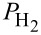 ≈ 5 × 10^−7^ Torr) on the chemical composition of a PtCl_2_ deposit as measured by AES. The initial deposit is composed almost exclusively of Pt and Cl, with a Pt/Cl ratio of 0.8. With increasing AH exposure, the Cl signal decreases monotonically until after 1.3 × 10^4^ L [51] of atomic hydrogen exposure, where the Pt/Cl ratio increased to 5.2. The growth of a small carbon peak observed at 273 eV is likely due to adventitious carbon deposited from the AH source. At this stage, the deposit was exposed to Ar^+^ sputtering. In addition to the loss of adventitious carbon, the Cl signal increased, causing a concomitant decrease in the Pt/Cl ratio from 5.2 to 2.8.

This sputtered deposit was subsequently re-exposed to AH, which again led to a decrease in the Cl signal. After an additional 7.2 × 10^3^ L AH exposure, the AES was dominated by Pt (Pt/Cl 10:1. Another period of Ar^+^ sputtering led to another significant increase in the Cl signal (Pt/Cl ratio of 2.2).

### Effect of atomic oxygen (AO) on FEBID deposits created from MeCpPtMe_3_

**Figure 7:**
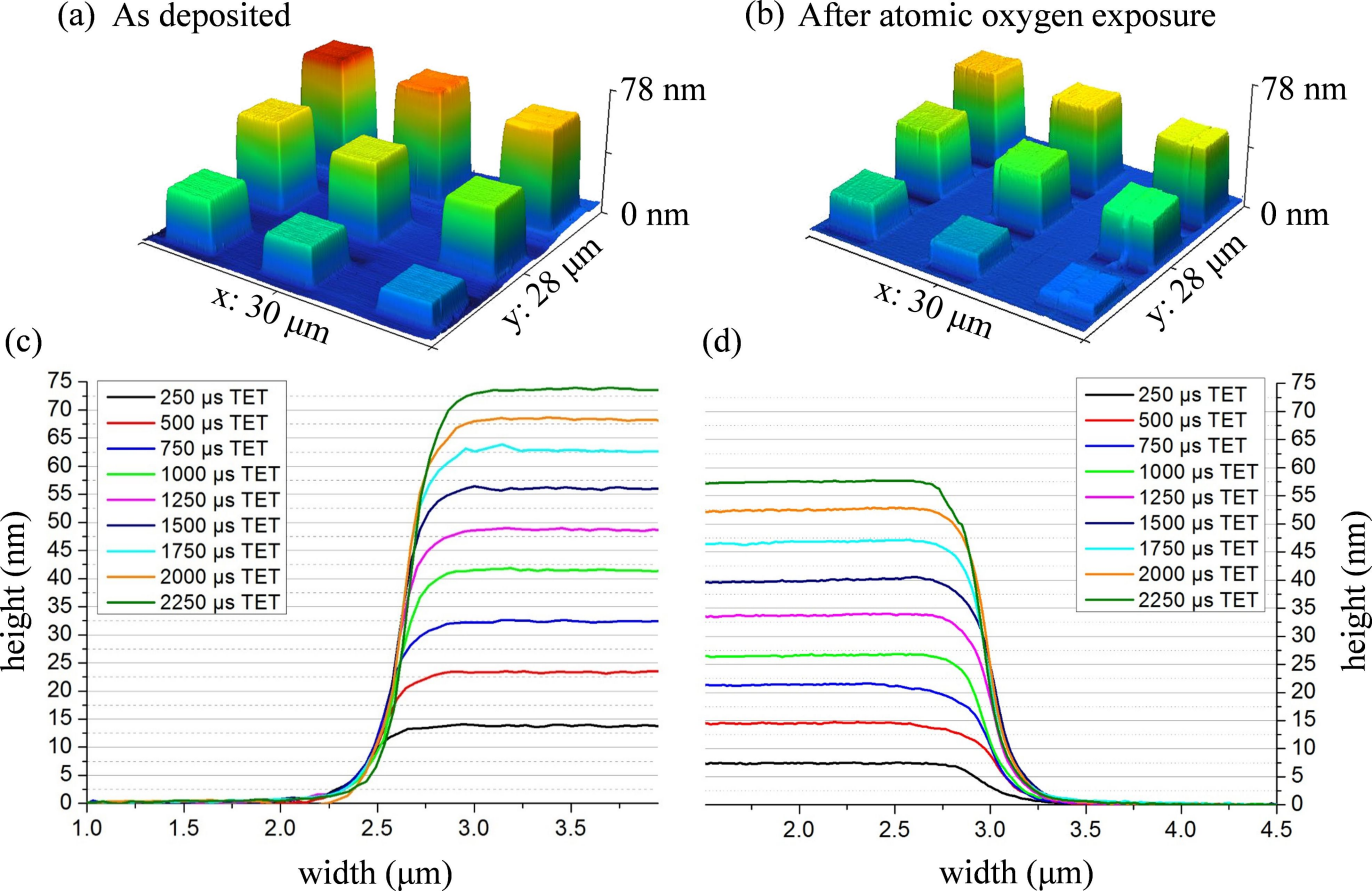
AFM data for nanostructures created from MeCpPtMe_3_ using different total exposure times (TET) (per pixel), before (left-hand side) and after (right-hand side) atomic oxygen exposure; (a) and (b) show AFM images of the three-dimensional pillars before and after atomic oxygen exposure, while (c) and (d) show the corresponding pillar heights.

Previous studies have shown that FEBID structures created from MeCpPtMe_3_ contain platinum atoms embedded in a carbonaceous matrix [7,44], which we will refer to hereafter as a PtC*_x_* structure. To produce PtC*_x_* FEBID deposits with well-defined shapes suitable for AFM analysis, a focused electron beam in combination with patterning was used to create a range of different deposits that exhibited the same (square) two-dimensional footprint. Figure 7a demonstrates that by controlling the deposition conditions, the height of the PtC*_x_* deposits can be systematically varied from 14–73 nm. Figure 7c shows AFM cross sectional profiles of nine FEBID PtC*_x_* pads with different total exposure times (TET) per pixel. Following a prolonged exposure to atomic oxygen, AFM analysis of the FEBID structures (Figure 7b,d) clearly shows that each of the PtC*_x_* structures has decreased in height.

**Figure 8:**
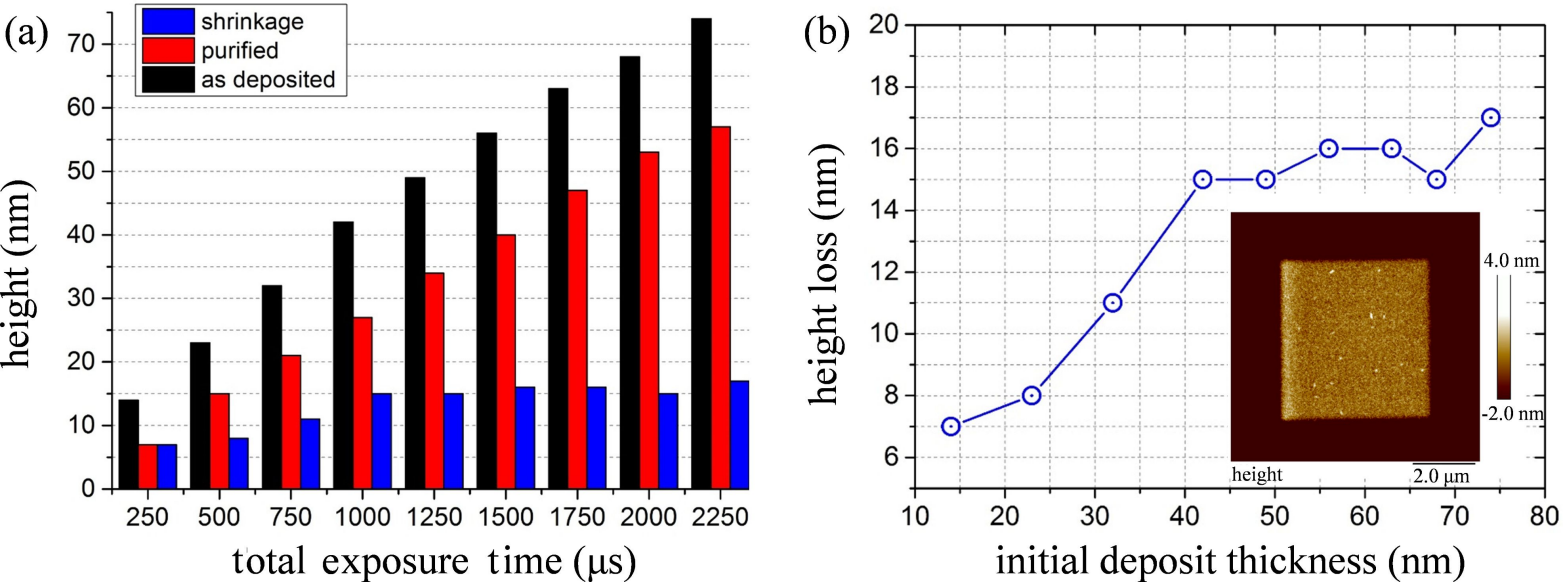
(a) Height of nanostructures created from MeCpPtMe_3_ as determined by AFM: as-deposited (black bar) and after exposure to atomic oxygen (red bar); the loss of height (or shrinkage) upon exposure to atomic oxygen is shown as the blue bar. (b) The height loss in the PtC*_x_* structures as a function of their initial thickness. The inset shows a top-down AFM image of one of the purified nanostructures.

Figure 8a compares the heights of the as-deposited (black) and purified (red) PtC*_x_* structures, plotted for each of the different deposits. As the height of the initial deposit increased from 15–40 nm so did the magnitude of the shrinkage (blue), ranging from 7 to 15 nm, respectively. In contrast, for deposits with an initial thickness in excess of 40 nm, the height loss after exposure to AO remains roughly constant at a value of 15–17 nm (Figure 8b). Figure 8b also shows an illustrative AFM image of a top-down view of a deposit after purification. The surfaces of the deposits are found to be slightly rougher than before purification but still extremely flat (rms roughness values ranging from 0.36–0.45 nm) and compact with no evidence of cracking or lateral shrinkage.

## Discussion

### Electrons

**Scheme 1:**
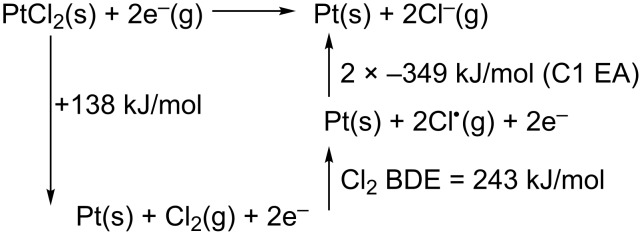
Hess cycle for electron purification of PtCl_2_. ∆*H* = −∆*H*_f_ PtCl_2_ + Cl_2_ bond dissociation enthalpy (BDE) + 2 × Cl electron affinity (EA) and therefore ∆*H* = 138 kJ/mol + 243 kJ/mol + 2 × (−349 kJ/mol) = −317 kJ/mol.

In sufficiently thin PtCl_2_ deposits, such as the one discussed in Supporting Information File 1, Figure S1b, electron beam postpurification can produce nearly pure Pt deposits (metal content increased from ≈40% to ≈87%). This is qualitatively consistent with our previous low-temperature UHV surface science studies on the effect of electron irradiation on 1–2 mL *cis*-Pt(CO)_2_Cl_2_ films [29]. Based on earlier studies [29,31] it is reasonable to assume that the purification process (PtCl_2_ + 2e^−^ → Pt(s) + 2 Cl^−^(g)) is initiated by low energy secondary electrons produced as a result of the interaction of the primary beam with the substrate. The ability of electrons to purify PtCl_2_ deposits by such an electron-stimulated process is also supported by the Hess cycle in Scheme 1 [52–53]. These calculations are consistent with an exothermic electron-stimulated purification process (−317 kJ/mol), driven principally by the electron affinity (EA) of chlorine (−349 kJ/mol [52]).

Figure 1 shows, however, that for thicker deposits (≈4× longer deposition time as compared to Supporting Information File 1, Figure S1b), the effect of postdeposition electron exposure is different. Specifically, although the Pt content in the near surface region (as determined by AES, see Figure 1b) initially increases upon electron exposure, after 7 h the Pt content remained constant at ≈55% (see Supporting Information File 1, Figure S1a) and was invariant to further increases in electron dose. In related studies, EDS of PtCl_2_ deposits exposed to 20 h of electron irradiation in the AES revealed only a small decrease (≈5%) in the Cl EDS signal (see Figure 1d). Similarly, electron beam irradiation of PtCl_2_ deposits in the SEM for 2 h at both 10 keV and 20 keV (data not shown) produced no detectable change in the Pt/Cl ratio measured by EDS. Considered collectively, these EDS and AES results on PtCl_2_ films of different thicknesses indicate that the loss of Cl^−^ ions is restricted to PtCl_2_ species in the nearest surface layers, on the same order of magnitude as the escape depth of the Auger electrons (inelastic mean free path = 1–2 nm [51,54]). However, Cl^−^ ions are almost certainly being produced by low energy secondary electrons, which are themselves being generated at significantly greater depths within the deposit (the depth of penetration of a 10 keV beam in PtCl_2_ is estimated to be ≈200 nm [50,55] based on a nominal PtCl_2_ density of 6.15 g/cm^3^ [56]). Consequently, our experimental observations suggest that the chloride ions generated in the deposit diffuse only very small distances (on the order of a few nanometers) before undergoing collision-induced charge neutralization and recombination with Pt. This scenario would lead to a minimal change in the EDS, as observed in Figure 1b,d because EDS measures the composition as defined by the depth of penetration of the primary beam (>100 nm) [55]. In contrast, the near surface region (topmost few nanometers) would ultimately reach a steady state composition when the rate of chlorine diffusion from deeper in the deposit into the near surface region is balanced by the rate of Cl loss due to electron-stimulated desorption. This is consistent with our AES results for thicker PtCl_2_ deposits (see Figure 1). Based on this mechanism, it would therefore be possible to remove all the Cl from a deposit, although the duration of electron irradiation would be prohibitive for all but the thinnest deposits.

Overall, postdeposition electron beam processing appears to be a viable strategy to remove halogens from sufficiently small/thin (a few nanometers) FEBID structures although the effect of purification on the structural integrity of the deposits still needs to be addressed. However, for thicker deposits/films, the effectiveness of postdeposition electron beam processing appears to be compromised not by the depth of electron penetration, but by the effective escape depth of the reactive chloride species formed. For larger FEBID structures, it should be noted that electron beam purification of nanostructures created from *cis*-Pt(CO)_2_Cl_2_ could still be effective if it is conducted in situ, under precursor limited deposition conditions. In such a scenario, each PtCl_2_ moiety deposited at the surface of the growing nanostructure could be exposed to a sufficiently large electron dose to effect Cl loss.

### Atomic hydrogen (AH)

**Scheme 2:**
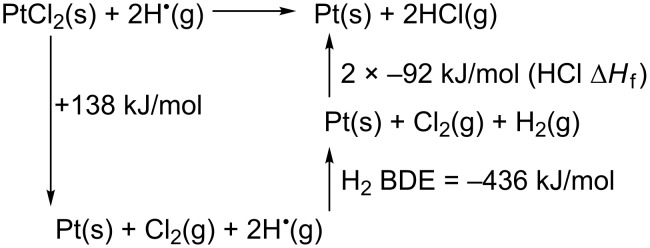
Hess cycle for atomic hydrogen purification of PtCl_2_. ∆*H* = −∆*H*_f_ PtCl_2_ + H_2_ bond dissociation enthalpy (BDE) + 2 × ∆*H*_f_ HCl and therefore ∆*H* = 138 kJ/mol − 436 kJ/mol + 2 × (−92 kJ/mol) = −482 kJ/mol.

As shown in Figures 2–5, exposure of PtCl_2_ deposits to AH using the high-pressure source resulted in complete chlorine removal for almost all of the deposits, regardless of initial thickness. As with electron irradiation, AH-mediated purification of PtCl_2_ deposits is thermodynamically viable. Indeed, the Hess cycle in Scheme 2 shows that the purification process is highly exothermic, driven principally by the innate reactivity of AH, coupled with the comparatively low formation enthalpy (∆*H*_f_) of PtCl_2_.

In addition to purification, exposure of the PtCl_2_ deposits to AH also frequently resulted in significant structural modifications to the deposits. In some instances these changes were fairly modest (Figure 2), but more extensive structural transformations were observed in the samples depicted in Figures 3–5. In general these structural transformations can be characterized by an increase in porosity and the formation of a honeycomb-like structure, although in one instance (Figure 4), AH purification leads to a complete loss of structural integrity in the purified Pt deposit. In Figure 5 the “cracking” in the deposits occurs exclusively in regions where the Cl atom concentration has significantly decreased (i.e., in regions of purification). These structural changes indicate that exposure of the PtCl_2_ deposits to AH and the resulting purification can also lead to significant Pt atom mobility. We believe that the mobility of Pt atoms can be rationalized in the context of scanning tunneling microscopy observations made by Horch et al., who found that the diffusion of under-coordinated Pt–H species was more than 500 times that of the same under-coordinated Pt atoms in the absence of adsorbed hydrogen [57]. Temperature programmed desorption data have shown that adsorbed H atoms desorb from Pt as H_2_ around room temperature [58]. Consequently, during AH purification, the steady state concentration of these mobile Pt–H species is determined by the following reaction sequence:


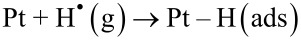



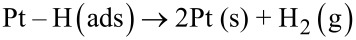


Thus, the concentration of Pt–H species will be directly proportional to the AH flux. Once Pt–H species are formed they can diffuse and nucleate with other Pt atoms or Pt–H species, although this mobility will cease once the AH source is turned off and the adsorbed H atoms desorb as H_2_. In Figures 3–5 we believe that we are observing the macroscopic manifestation of enhanced Pt atom mobility, facilitated by the large flux of AH. The formation of a porous structure is believed to be a consequence of the increased mobility of under-coordinated Pt atoms as seen by Horch [57]. However, once these under-coordinated Pt atoms diffuse and nucleate, they will become integrated into larger ensembles of Pt atoms where their enhanced mobility will be lost. Under these circumstances, a porous structure would be expected to form. An analogous mechanism has been previously proposed to account for the formation of stochastic patterns of different sized clusters on surfaces [59].

Most likely, the differences in morphological changes observed for different PtCl_2_ deposits exposed to AH are caused by variations in the AH flux between different experiments, which would not be expected to affect the overall ability of AH to purify PtCl_2_ deposits, but would change the steady state concentration of Pt–H species and thus the nature and extent of the structural transformation. Pt atom diffusion could be further enhanced by the significant exothermicity of the purification reaction (PtCl_2_(s) + 2H(ads) → Pt(s) + 2HCl(g); Δ*H* = −482 kJ/mol) which could increase the local temperature within the deposit.

In contrast to electron-mediated purification (Figure 1), film thickness did not affect the ability of AH to remove chlorine from PtCl_2_ (Figures 2–4). This indicates that AH was able to diffuse (and therefore react) throughout the deposit. In part the ease of hydrogen atom diffusion within the deposit can be ascribed to its extremely small size (atomic radius = 37 pm [52]). The porosity of the purified Pt structures will also facilitate AH diffusion throughout the entire deposit and also to the ease with which gas phase species, such as HCl, produced in the purification process can desorb from the deposit.

Studies conducted with the low-pressure atom source (Figure 6) demonstrate that AH purification occurs in a top-down process, propagating from the surface into the bulk. Thus, the AES data in Figure 6 show that the Cl atom concentration in the near surface region decreases systematically as the AH exposure increases. However, when the AH-treated samples are Ar^+^ ion sputtered, the Cl content increases, revealing the presence of unreacted PtCl_2_ below the topmost few nanometers of material analyzed by AES. The top-down nature of the purification process is expected given that purification in the bulk of the deposit requires AH diffusion, which will lead to a depth-dependent concentration gradient of AH within the deposit. In the higher pressure AH studies, the directional nature of the purification is obscured by AH flux several orders of magnitude larger.

The increased porosity during AH purification of PtCl_2_ is in sharp contrast to the densification observed during electron beam purification of PtC*_x_* and AuC*_x_* deposits using electron beam irradiation in the presence of either oxygen or water [13,17,60]. One other interesting observation is the general absence of structural transformations in regions of PtCl_2_ deposits that were initially exposed to electron irradiation in the SEM in order to acquire EDS data prior to AH exposure (see Figure 3 and Supporting Information File 1, Figure 2). It should be noted that the location of these EDS analysis regions could not be ascertained visually prior to AH exposure and the resultant purification of the PtCl_2_ deposits. The cause of this structural transformation is unclear, but one possibility is that the electron beam exposure required to acquire EDS data was sufficient to purify the very topmost layer of the PtCl_2_ deposits by electron-stimulated desorption of Cl^−^ anions, although this would not be expected to change the Pt/Cl ratio measured by EDS. The Pt atoms in this region could be less susceptible to the effects of subsequent AH exposure, possibly due to Pt nucleation and coalescence in the electron beam purification step and/or a decrease in local heating from the exothermicity of the AH purification step (which would enhance the mobility of Pt–H species) as compared to the rest of the PtCl_2_ structure. Another possible explanation is that carbonaceous deposits, which are more resistant to subsequent AH etching, are formed in regions of the PtCl_2_ deposit initially exposed to electrons during EDS analysis.

In summary, AH is extremely effective at removing chlorine from PtCl_2_ and creating pure Pt deposits, regardless of the thickness of the deposit. However, in sharp contrast to the densification observed during electron beam purification strategies [13,17,21], AH-mediated purification of PtCl_2_ typically leads to the creation of highly porous Pt structures, and in some instances, to a loss of structural integrity, neither of which are desirable for FEBID structures. These porous structures could, however, have interesting applications, for example, as high surface area catalysts. This phenomenon of AH-mediated dispersion could also be exploited to help redisperse metals, such as Pt, which have nucleated as a result of sintering during catalysis.

### Other Pt-containing deposits

In contrast to the effectiveness of AH towards purification of PtCl_2_, there was no measureable removal of carbon or phosphorus contaminants for FEBID structures created from MeCpPtMe_3_, Pt(hfac)_2_, and Pt(PF_3_)_4_, and no significant morphological changes in the deposits (see Table 1 and Supporting Information File 1, Figures S4–S6). In the scientific literature that shows that AH can chemically etch carbon [61–62] (for example, Botman et. al. [26]) a decrease in carbon content is observed from 81% to 65% upon exposing the PtC*_x_* deposits to a high-pressure AH source. However, the current work demonstrates that the efficiency of AH-mediated carbon and phosphorus purification is significantly lower than that of halogen atom removal. This difference in efficiency may be attributed to the number of elementary steps required to generate a stable gas-phase species from carbon (C(ads) + H(g) →→ CH_4_(g)) or phosphorus (P(ads) + H(g) →→ PH_3_(g)), coupled with the reversibility of AH addition/abstraction reactions (e.g., CH_2_(ads) + H(g) 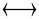 CH_3_(ads); CH_2_(ads) + H(ads) 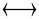 CH(ads) + H_2_(g)) [61–62]. In contrast, halogen atom removal by AH requires a single irreversible reaction, generating a volatile product (X(ads) + H(g) → HX(g)).

### Atomic oxygen

Atomic oxygen is known to react with carbon to form volatile CO and CO_2_ species [18,63] in an energetically favorable process (e.g., C(ads) + O(g) → CO(g): Δ*H* = −359 kJ/mol [52]). Results from the present study can be most directly compared with a postdeposition study by Plank et al. [17], where electron beam processing in the presence of an O_2_ flux was used to create reactive oxygen species (most likely AO) for purification of FEBID structures created from MeCpPtMe_3_. In both studies, densification of the topmost layers of the structures was observed as the deposits were purified, evidenced in the present study by the decrease in the height of the deposits after AO exposure. Interestingly, the observation of densification in the present study suggests that this phenomenon does not necessarily require electron irradiation.

For the present study, the height loss plateau at ≈15 nm is an indication of the limited penetration depth of the AO within the deposits. In the work of Plank et al., a similar top-down purification process with a limited penetration depth was observed where the purification depth was determined principally by the effective diffusion length of O_2_, evidenced experimentally by an increase in purification depth as the partial pressure of oxygen increased. Similarly, we expect that AO will also have limited diffusion within the deposit. Indeed, given the inherent reactivity of AO and the potential for AO recombination reactions (O + O → O_2_) we would expect AO to have a small penetration depth within the deposit. This is at least qualitatively consistent with our experimentally determined purification depth of ≈15 nm, while the lowest value observed in the O_2_/e^−^ process [17] was on the order of 50 nm, using a similar 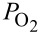 to that used in the present study. Previous studies [17,21] have noted that as top-down purification occurs, there is a densification of the top layer of the deposit, which may also reduce AO diffusion into the bulk. It should be noted that larger fluxes of AO, for example, those generated by an atmospheric plasma [64], will almost certainly increase the depth of purification, paralleling the effect of O_2_ pressure in the O_2_/e^−^ purification process. However, in either case, the overall effectiveness is limited by the diffusion of the oxygen-containing species, an issue that is overcome when electron beam purification is performed using water vapor [13,23,60].

The contrast between the effect of AO on PtC*_x_* deposits and AH on PtCl_2_ is striking. On one hand, AH is able to completely purify PtCl_2_ deposits without any evidence of a limited purification depth. In part this can be ascribed to the smaller size of AH (atomic radius H = 37 pm [52]) compared to AO (atomic radius O = 66 pm [52]), but is more likely governed principally by the effect of purification on the structure of the deposits. In the case of AO reactions with PtC*_x_*, the deposits become more compact as C atoms are removed, which will decrease the penetration depth of the AO. In contrast, AH reactions with PtCl_2_ deposits lead to a more open and porous structure that will facilitate penetration of gas phase species. The qualitatively similar enthalpy of reaction for carbon atom removal by AO (≈−394 kJ/mol) [52] to Cl atom removal by AH purification (≈−483 kJ/mol) further supports the idea that the enhanced Pt atom mobility in the presence of AH is due principally to a chemical as opposed to a physical (e.g., heating) phenomenon.

## Conclusion

Contaminant Cl atoms can be removed by postdeposition processing of PtCl_2_ FEBID structures created from *cis*-Pt(CO)_2_Cl_2_ using electrons or atomic hydrogen. However, the effectiveness of these two species with respect to chlorine atom removal differs markedly. In the case of electrons, chloride ions are only removed from PtCl_2_ present in the near surface region (1–2 nm depth). This process would thus be viable only for small/thin (few nanometers) FEBID structures. For atomic hydrogen, the purification process is efficient and not limited to a surface reaction. This difference is ascribed in large part to the porosity of the purified structure generated by AH, ascribed to the transient formation of mobile Pt–H species during the purification process. The mobility of these Pt–H species leads to significant structural transformations which are detrimental for FEBID applications, but potentially useful in reversing the effects of sintering in catalysis and in creating high surface area catalysts. The purification of PtC*_x_* deposits created from MeCpPtMe_3_ using AO was found to exhibit many of the same characteristics of postdeposition purification using electron beam irradiation in the presence of oxygen, including densification and a limited purification depth.

## Supporting Information

File 1 Additional experimental information.
Percent platinum content as a function of electron beam irradiation for a PtCl_2_ deposit and Auger spectra detailing the effects of electron beam irradiation on a relatively thin PtCl_2_ deposit; reference EDS data for the substrate, Pt foil, and a typical PtCl_2_ deposit before and after atomic hydrogen purification; SEM and EDS data for a PtCl_2_ deposit exposed to 10 min of atomic hydrogen treatment; AFM analysis of the edge of a PtCl_2_ deposit after loss of structural integrity; SEM and EDS data for FEBID deposits created from MeCpPtMe_3_ and Pt(hfac)_2_, before and after atomic hydrogen treatment; SEM and WDS data for a FEBID deposit created from Pt(PF_3_)_4_, before and after atomic hydrogen treatment.
