# Supplementary material for: Comparing postdeposition reactions of electrons and radicals with Pt nanostructures created by focused electron beam induced deposition
Source: Beilstein J Nanotechnol. 2017 Nov 15;8:2410–24. doi: 10.3762/bjnano.8.240 (PMC5704761; doi:10.3762/bjnano.8.240)
Supplement: File 1 — Additional experimental information. Percent platinum content as a function of electron beam irradiation for a PtCl2 deposit and Auger spectra detailing the effects of electron beam irradiation on a relatively thin PtCl2 deposit; reference EDS data for the substrate, Pt foil, and a typical PtCl2 deposit before and after atomic hydrogen purification; SEM and EDS data for a PtCl2 deposit exposed to 10 min of atomic hydrogen treatment; AFM analysis of the edge of a PtCl2 deposit after loss of structural integrity; SEM and EDS data for FEBID deposits created from MeCpPtMe3 and Pt(hfac)2, before and after atomic hydrogen treatment; SEM and WDS data for a FEBID deposit created from Pt(PF3)4, before and after atomic hydrogen treatment. [file Beilstein_J_Nanotechnol-08-2410-s001.pdf]

Supporting Information

for

Comparing postdeposition reactions of electrons and radicals with Pt nanostructures created by focused electron beam induced deposition

Julie A. Spencer[1], Michael Barclay[1], Miranda J. Gallagher[1], Robert Winkler[2], Ilyas Unlu[1], Yung-Chien Wu[3], Harald Plank[2,4], Lisa McElwee-White[3], and D. Howard Fairbrother[1,*]

Address: [1]Department of Chemistry, Johns Hopkins University, Baltimore, MD, 21218, USA, [2]Graz Centre for Electron Microscopy, Steyrergasse 17, 8010 Graz, Austria, [3]Department of Chemistry, University of Florida, Gainesville, FL, 32611-7200, USA, and [4]Institute of Electron Microscopy and Nanoanalysis, Graz University of Technology, Steyrergasse 17, 8010 Graz, Austria

[*]Corresponding Author

Email: D. Howard Fairbrother - howardf@jhu.edu

Additional Experimental Information

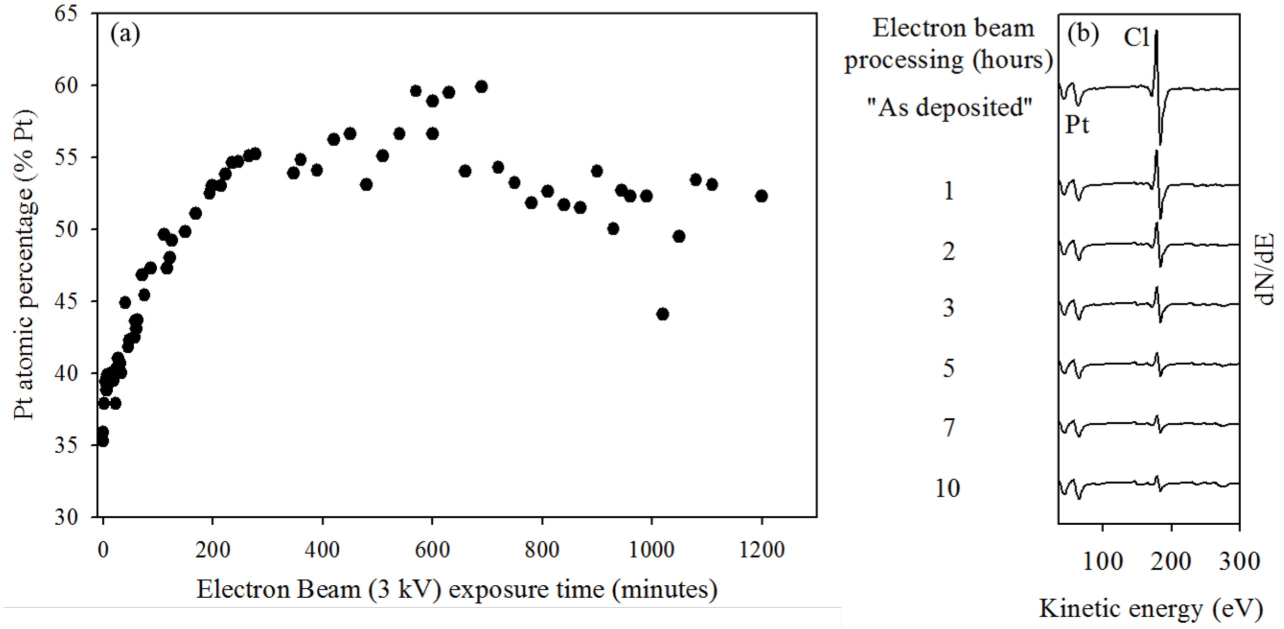

Figure S1: (a) Pt atomic percentage (% Pt) for the $PtCl_2$ deposit shown in Figure 1, plotted as a function of electron beam irradiation. (b) Evolution of Auger spectra for a thinner deposit (5.25 hours deposition compared with 22 hours deposition) created from *cis*-$Pt(CO)_2Cl_2$ exposed to electron beam irradiation.

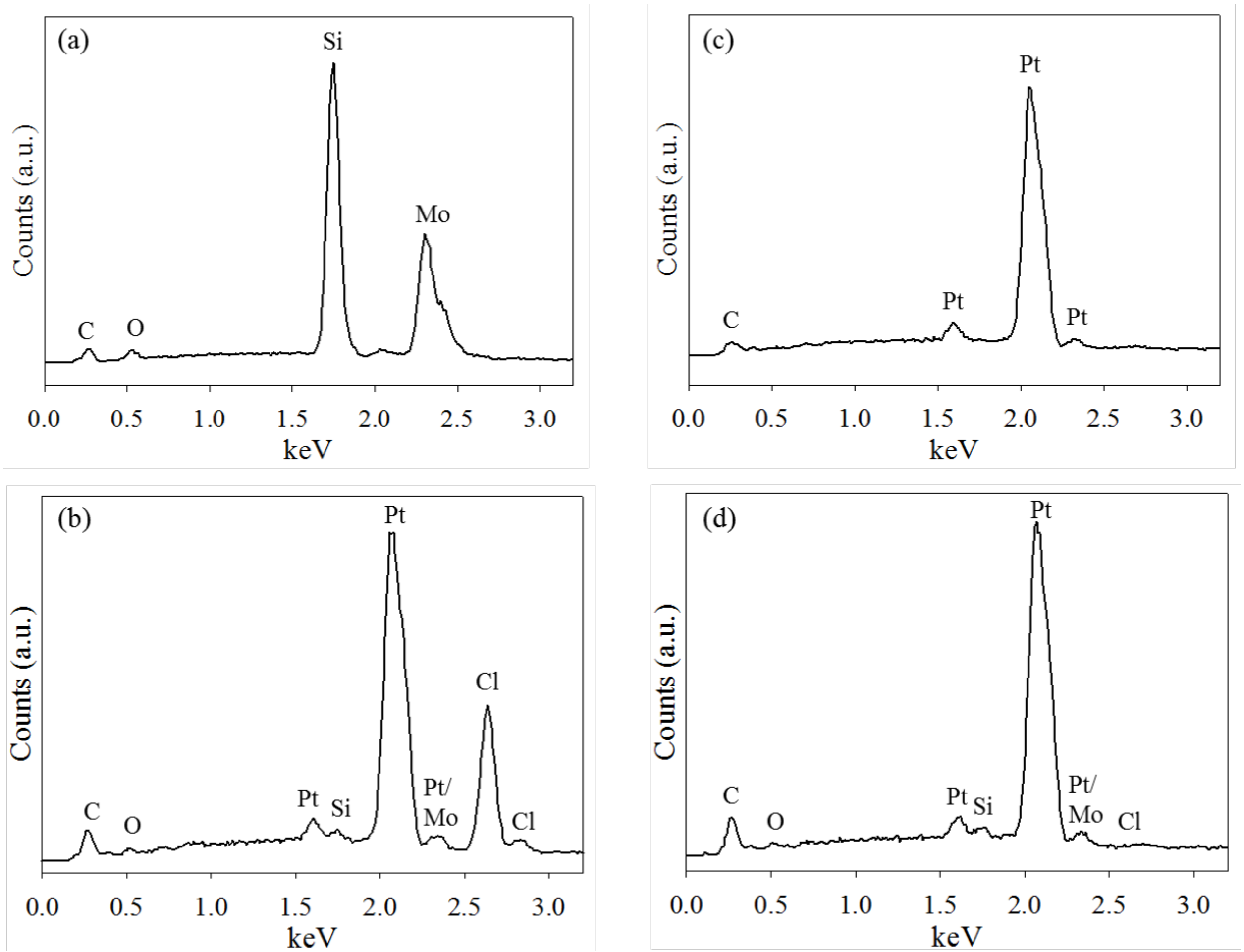

Figure S2: Reference EDS data are shown for (a) Ru coated Si/Mo multi-layer mirror substrate, (b) an as-deposited $PtCl_2$ deposit, (c) reference pure Pt foil and (d) a purified $PtCl_2$ deposit after the majority of Cl has been removed by atomic hydrogen processing.

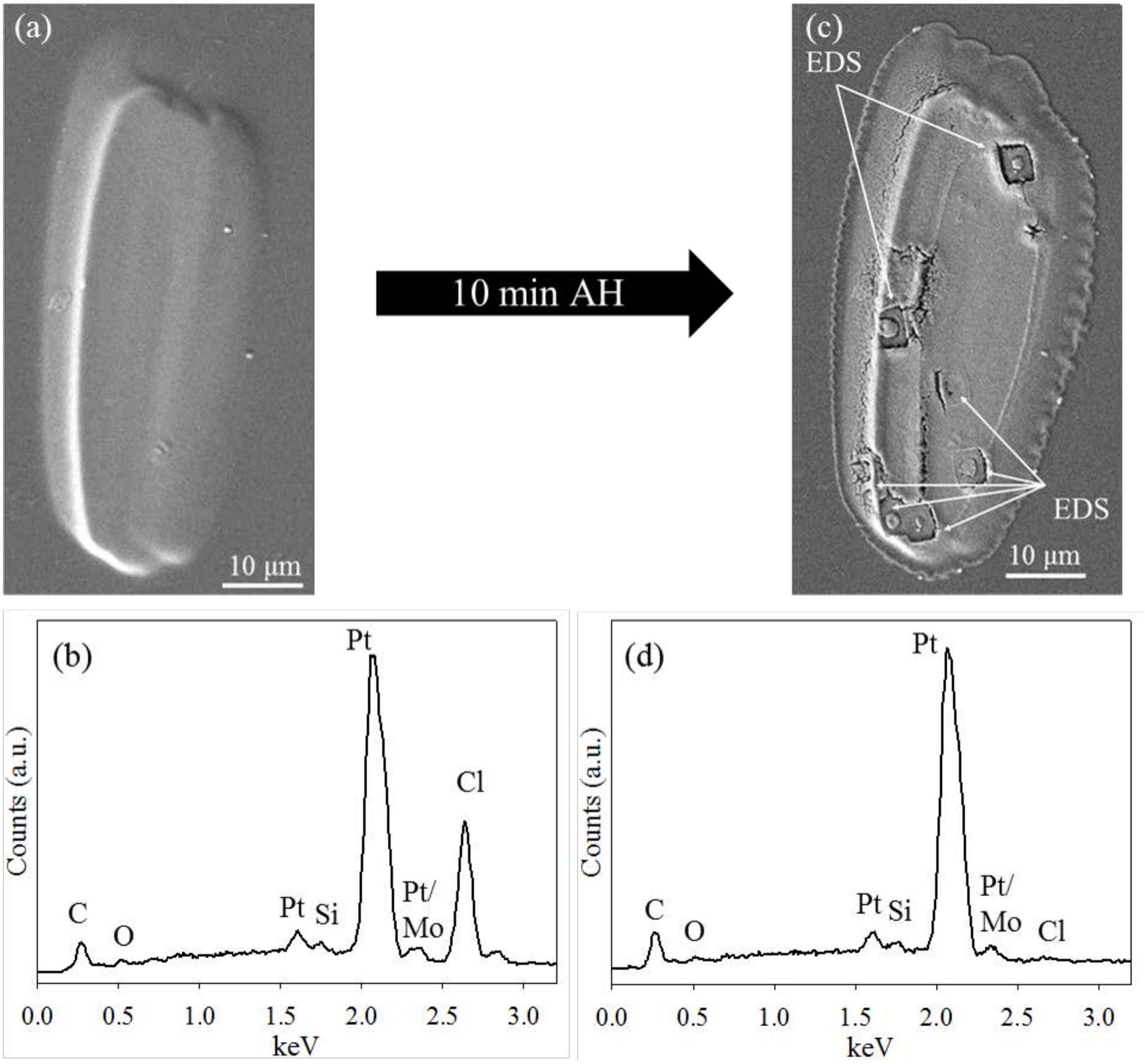

Figure S3: (a) SEM image of a deposit created from *cis*-$Pt(CO)_2Cl_2$, and (c) after exposure to 10 minutes of atomic hydrogen treatment at P_{H_2} ~1 Torr (6.0 x 10^8 L). The EDS spectra for this deposit before and after AH exposure are shown in (b and (d); areas where the deposit was exposed to EDS analysis prior to AH exposure are marked with arrows in (c).

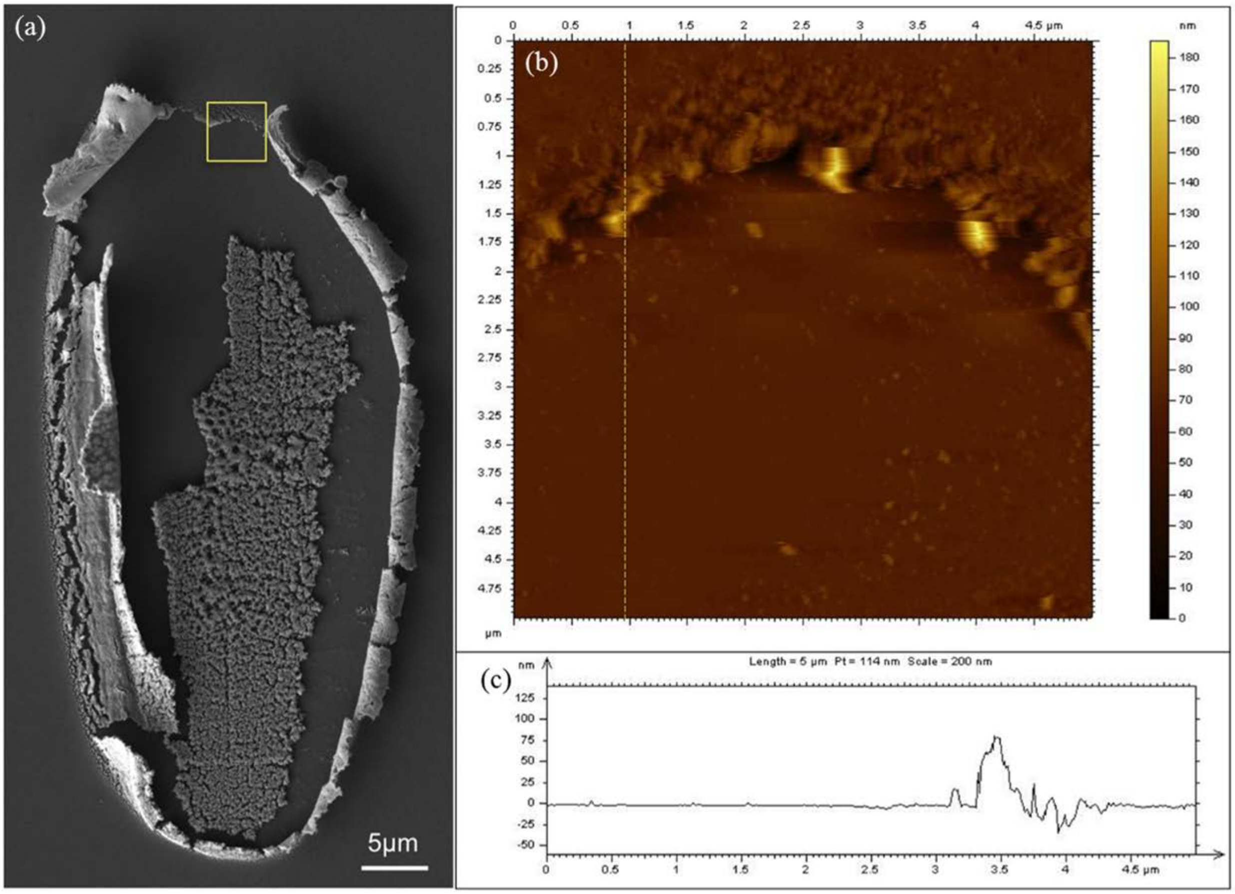

Figure S4: (a) Composite SEM image at higher magnification of $PtCl_2$ deposit shown in Figure 4. After 4 hours of atomic hydrogen treatment (P_{H_2} ~ 1 Torr, 7.2 x 10^9 L), significant morphological changes occurred. AFM height profile data from inside to outside within the yellow square was determined to be 100 nm as shown in (b) the 5 μm^2 AFM topography image and in (c) profile extraction across x = 0.9 μm.

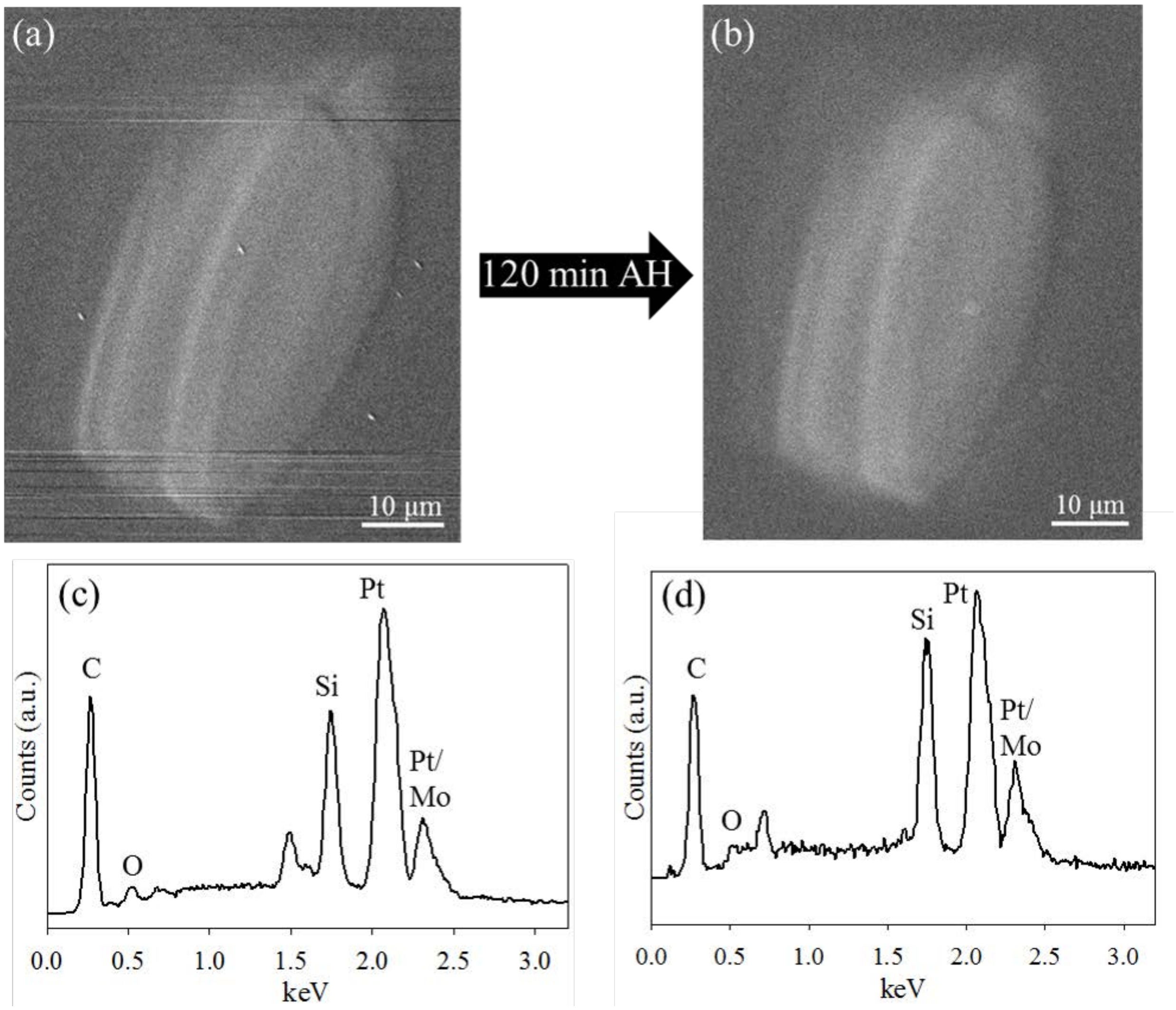

Figure S5: SEM and EDS data for a deposit created from $MeCpPtMe_3$ on a Ru coated Si/Mo multi-layer substrate. A SEM image of this deposit prior to atomic hydrogen treatment is shown in (a), with the corresponding EDS spectra shown in (c). The deposit is shown in (b) after 2 hours of atomic hydrogen treatment at P_{H_2} ~ 1 Torr (7.2 x 10^9 L), and corresponding EDS data is shown in (d).

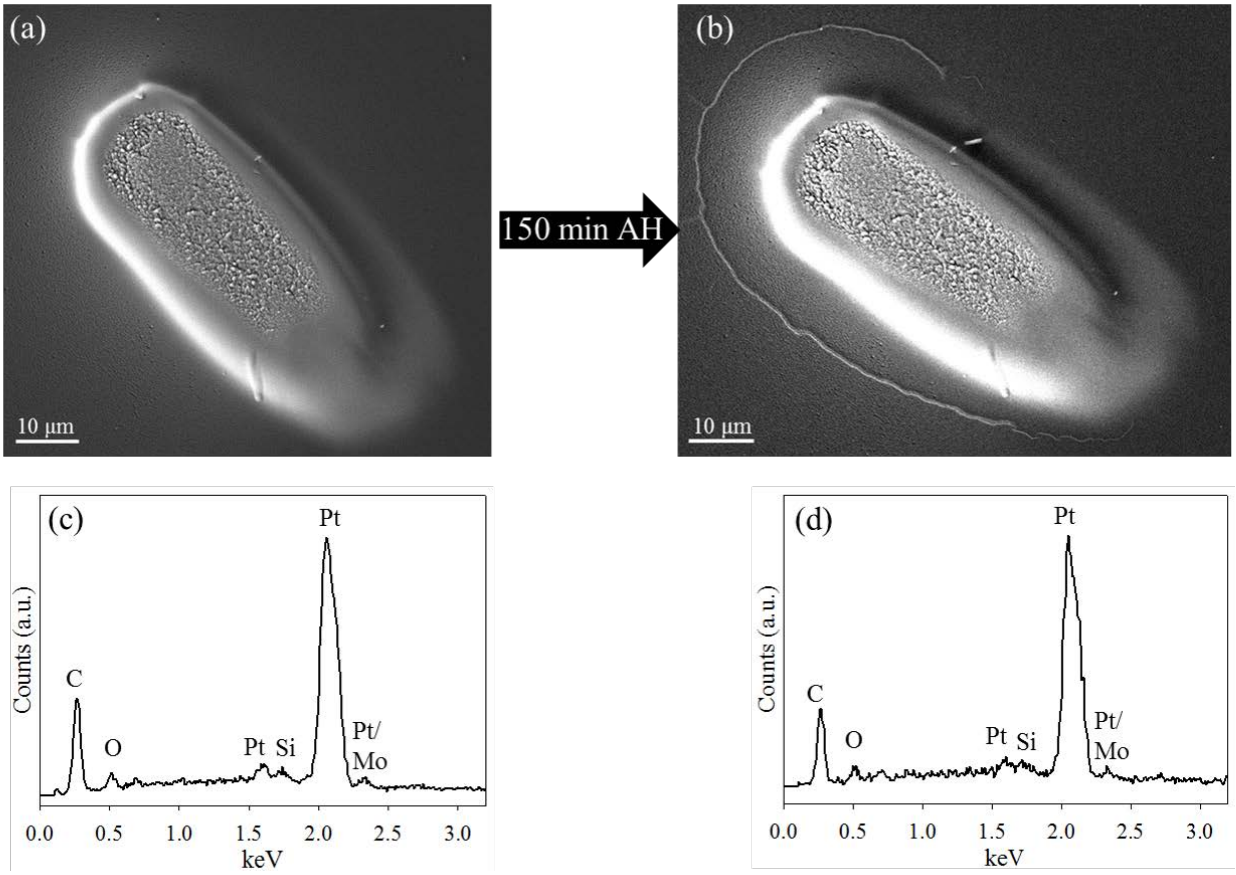

Figure S6: SEM and EDS data for a deposit created from $Pt(hfac)_2$ on a SiO_2 substrate. A SEM image of this deposit prior to atomic hydrogen treatment is shown in (a), with the corresponding EDS spectra shown in (c). The deposit is shown in (b) after 2.5 hours of atomic hydrogen treatment at P_{H_2} ~ 1 Torr (9.0 x 10^9 L), and corresponding EDS data is shown in (d).

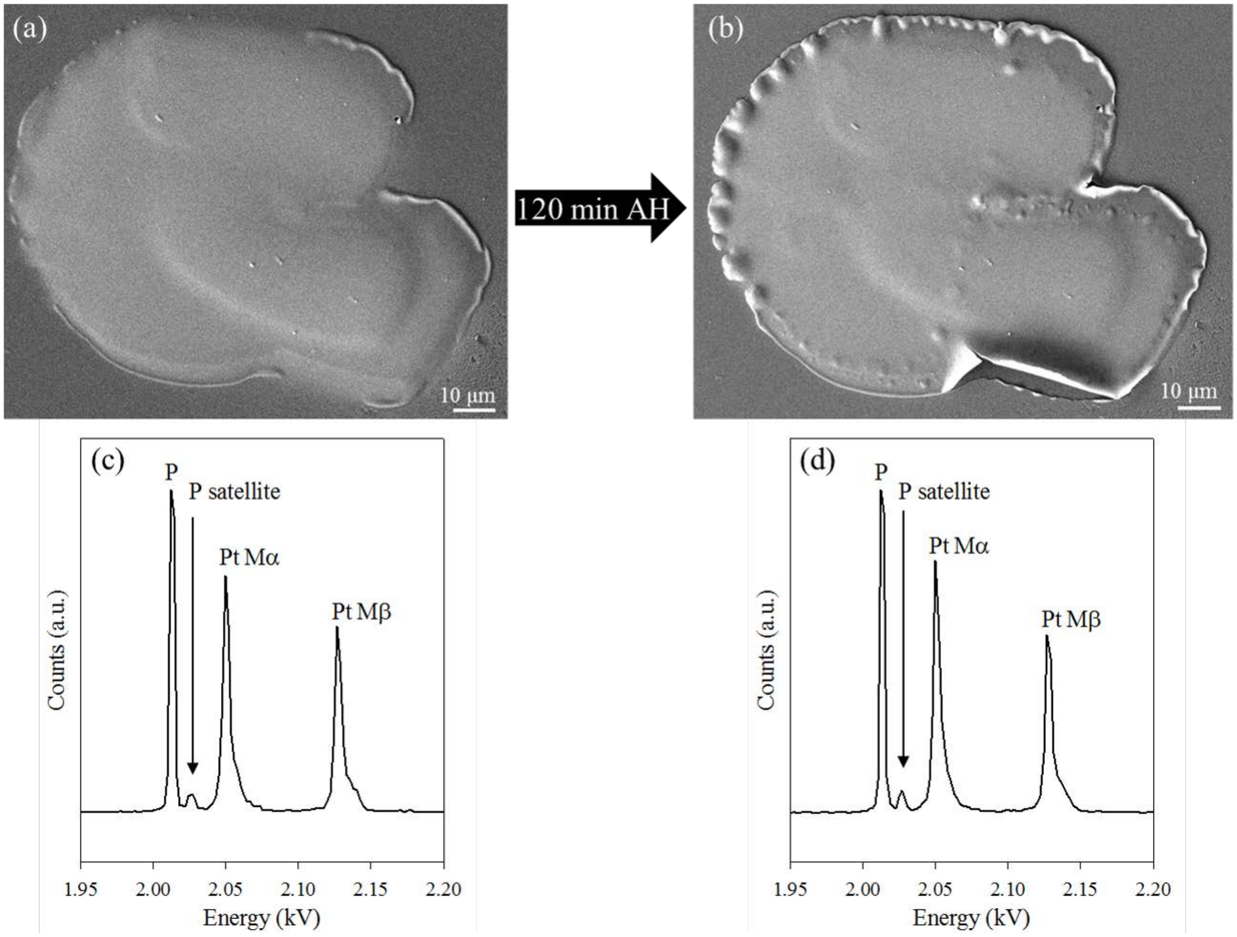

Figure S7: SEM and WDS data for a deposit created from $Pt(PF_3)_4$ on a Ru coated Si/Mo multi-layer substrate. A SEM image of this deposit prior to atomic hydrogen treatment is shown in (a), with (c) the corresponding WDS spectra. The deposit is shown in (b) after 2 hours of atomic hydrogen treatment at P_{H_2} ~ 1 Torr (7.2 x 10^9 L), and corresponding WDS data is shown in (d).
